# Transcriptome-wide analysis reveals the coregulation of RNA-binding proteins and alternative splicing genes in the development of atherosclerosis

**DOI:** 10.1038/s41598-022-26556-6

**Published:** 2023-01-31

**Authors:** Runqing Wang, Jin Xu, Yuning Tang, Yongxiang Wang, Jing Zhao, Liqiong Ding, Yu Peng, Zheng Zhang

**Affiliations:** 1 The First School of Clinical Medicine, Lanzhou University, Lanzhou, Gansu China; 2 Gansu Key Laboratory of Cardiovascular Diseases, The First Hospital of Lanzhou University, Lanzhou, Gansu China; 3 Gansu Clinical Medical Research Center for Cardiovascular Diseases, The First Hospital of Lanzhou University, Lanzhou, Gansu China; 4 Department of Cardiology, Lanzhou University Second Hospital, Lanzhou, China; 5 Heart Center, The First Hospital of Lanzhou University, Lanzhou, Gansu China

**Keywords:** Cardiology, Molecular medicine

## Abstract

RNA-binding proteins (RBPs) are involved in the regulation of RNA splicing, stability, and localization. How RBPs control the development of atherosclerosis, is not fully understood. To explore the relevant RNA-binding proteins (RBPs) and alternative splicing events (ASEs) in atherosclerosis. We made a comprehensive work to integrate analyses of differentially expressed genes, including differential RBPs, and variable splicing characteristics related to different stages of atherosclerosis in dataset GSE104140. A total of 3712 differentially expressed genes (DEGs) were identified, including 2921 upregulated genes and 791 downregulated genes. Further analysis screened out 54 RBP genes, and 434 AS genes overlapped DEGs. We selected high expression ten RBP genes (SAMHD1, DDX60 L, TLR7, RBM47, MYEF2, RNASE6, PARP12, APOBEC3G, SMAD9, and RNASE1) for co-expression analysis. Meanwhile, we found seven regulated alternative splicing genes (RASGs) (ABI1, FXR1, CHID1, PLEC, PRKACB, BNIP2, PPP3CB) that could be regulated by RBPs. The co-expression network was used to further elucidate the regulatory and interaction relationship between RBPs and AS genes. Apoptotic process and innate immune response, revealed by the functional enrichment analysis of RASGs regulated by RBPs were closely related to atherosclerosis. In addition, 26 of the 344 alternative splicing genes regulated by the above 10 RBPs were transcription factors (TFs), We selected high expression nine TFs (TFDP1, RBBP7, STAT2, CREB5, ERG, ELF1, HMGN3, BCLAF1, and ZEB2) for co-expression analysis. The target genes of these TFs were mainly enriched in inflammatory and immune response pathways that were associated with atherosclerosis. indicating that AS abnormalities of these TFs may have a function in atherosclerosis. Furthermore, the expression of differentially expressed RBPs and the alternative splicing events of AS genes was validated by qRT-PCR in umbilical vein endothelial cells (HUVEC). The results showed that RBM47 were remarkedly difference in HUVEC treated with ox-LDL and the splicing ratio of AS in BCLAF1which is regulated by RBM47 significantly changed. In conclusion, the differentially expressed RBPs identified in our analysis may play important roles in the development of atherosclerosis by regulating the AS of these TF genes.

## Introduction

Atherosclerosis is the accumulation of lipids in the intima of arteries and the formation of the core of atherosclerotic plaques. As the disease progresses, atherosclerotic plaques develop fibrous caps and gradually accumulate calcium minerals^1^. Late unstable atherosclerotic plaques rupture and cause thrombi, occluding blood vessels and causing acute ischemia of organs, while stable atherosclerotic plaques invade the lumen volume and cause chronic tissue ischemia^2^. Atherosclerosis can lead to a variety of cardiovascular diseases, such as myocardial infarction, ischemic cardiomyopathy, stroke, and peripheral arterial disease, of which heart disease and stroke are the two leading causes of death worldwide^3^. In 2016, more than 17 million people died of cardiovascular disease, accounting for 31% of global deaths^4^. At present, the treatment of atherosclerosis focuses on lipid-lowering, anti-platelet and late-stage vascular recanalization, and there are few measures related to how to prevent plaque progression^1^. Therefore, understanding the key physiological mechanisms of atherosclerosis progression is of great significance for preventing patients from developing corresponding clinical symptoms and improving patient prognosis.

Approximately 95% of human multiexon genes generate different transcripts through alternative splicing (AS), which leads to diversity in genetic information transmission^5^. AS processes play important roles in tissue recognition and many key biological processes^6^. To date, functional roles mediated by AS have been discovered in the fields of cardiac development, myocardial infarction, and structural cardiac diseases^7^. Through these studies, research has shown that AS plays an important role in atherosclerosis. For example, AS of FOXP3 can activate regulatory T cells (Treg cells) and inhibit the progression of atherosclerotic disease^8^. In ApoE (−/−) mice fed a high-fat diet, SRPK1-mediated splicing of VEGF-A to proangiogenic VEGF165 was found to contribute to the development of AS^9^. The binding of RNA-binding proteins (RBPs) through specific target sites can drive AS, but the changes in RBPs and AS that mediate atherosclerosis are currently unclear.

We explored RBPs and AS events (ASEs) in atherosclerosis using bioinformatics and expression analysis methods based on previously reported gene expression data of human fibroatheroma samples (GSE104140 dataset). The data were divided into the very early stage of vascular disease (diffuse intimal thickening) and two advanced stages of atherosclerosis (calcified and noncalcified fibroatheroma formation). In the first step, we identified differentially expressed genes (DEGs) in the dataset and then annotated and analyzed their functions. Moreover, we examined differentially prevalent ASEs and differentially expressed RBPs and found changes in AS at different stages and their potential regulatory functions in atherosclerosis, laying a foundation for the in-depth study of the molecular mechanism underlying the occurrence and development of atherosclerosis.

## Materials and methods

### Retrieving and processing data

We used “atherosclerosis” as the key word to retrieve and select appropriate datasets from the Gene Expression Omnibus (GEO) database and ultimately identified the GSE104140 dataset for use in further analyses. The GSE104140 dataset contains transcriptomic high-throughput sequencing data retrieved using a GPL16791 platform (Illumina HiSeq 2500 Homo sapiens). Total RNA was extracted from sets of thawed plaque cryosections. The expression profiles were converted and standardized into log2 data to create a series matrix file. GSE104140 is a transcriptome sequencing dataset of human fibroatheromas, which contains 32 samples; The 32 samples were all from carotid endarterectomy plaque sections with various pathological types. 4 samples with inconsistent pathological types were eliminated, including intimal xanthoma, pathologic intimal thickening (with superficial macrophages), and pathologic intimal thickening (macrophage poor). And the remaining 28 samples were allocated into 3 groups: diffuse intimal thickening (DIT) includes 8 biological replicates, calcified fibroatheromas include 12 biological replicates, and noncalcified fibroatheromas include 8 biological replicates.

### Read alignment and DEG analysis

We utilized TopHat2^10^ to align the reads to the human genome (GRch38) by setting the mismatch parameter to 4. Each gene was evaluated based on the number of reads and fragments per kilobase of exon per million mapped fragments (FPKM) using unique mapped reads. We utilized DEseq2^11^ to analyze all the DEGs in the dataset. We identified differentially expressed genes on the basis of fold change (FC ≥ 2 or ≤ 0.5) and false discovery rate (FDR ≤ 0.05) criteria.

### Differential AS analysis

Alternative splicing events (ASEs) and regulated ASEs (RASEs) between different groups were predicted and quantified based on previously reported methods^12,13^. Based on splicing junction reads, we finally detected ten types of ASEs, including alternative 5' splice site (A5SS), alternative 3' splice site (A3SS), cassette exon, exon skipping (ES), intron retention (IR), mutually exclusive exons (MXE), mutually exclusive 5' untranslated regions (UTRs; 5pMXE), mutually exclusive 3'UTRs (3pMXE), A3SS&ES and A5SS&ES data. We calculated the ratio of alternatively spliced to constitutively spliced reads as the RASE ratio between compared samples. We set a *p value *≦ 0.05 for RASE discovery. Student's *t test* was performed to assess an altered ASE ratio in a repetition comparison. The differences in ASEs at the p-value cutoff of 0.05 were considered RASEs.

### Functional enrichment analysis

To distinguish enriched functional pathways of DEGs, we performed a Gene Ontology functional enrichment analysis and Kyoto Encyclopedia of Genes and Genomes (KEGG) pathway enrichment analysis. Functional enrichment statistics of selected gene sets were predicted using GO and KEGG pathway annotation with the KOBAS 2.0 server^14^. For significant pathways, we used a hypergeometric test and Benjamini–Hochberg FDR control to determine the statistical enrichment of each pathway.

### Cell culture and treatment

We purchased human umbilical vein cells (HUVECs) from the American Type Culture Collection (ATCC; Manassas, VA, USA) and cultured them in endothelial cell medium (ScienCell, California, USA) with 5% (v/v) fetal bovine serum (FBS) (ScienCell, California, USA), 100 U/ml penicillin, 100 mg/ml streptomycin (ScienCell, California, USA), and 1% ECGF (ScienCell, California, USA). The cells were incubated in a 5% CO_2_ incubator at 37 °C. To mimic atherosclerosis in vitro, HUVECs were stimulated with 100 μg/ml ox-LDL (UnionBiol, Beijing, China) for 24 h.

### Quantitative real-time polymerase chain reaction (qRT–PCR)

Total RNA was extracted using an M5 HiPer Universal RNA Mini kit (Mei5bio, Beijing, China). The cDNA was reverse transcribed from total RNA by an M5 Super Plus qPCR RT kit with gDNA remover (Mei5bio, Beijing, China), and the reverse transcription reactions were performed on a Quant Studio™ Dx instrument (Thermo Fisher Scientific, USA) using HieffTM qPCR SYBR® Green (Yeasen, Shanghai, China). The qPCR reactions were run under the following conditions: 5 min at 95 °C for 1 cycle, 15 s at 95 °C and 30 s at 60 °C for 40 cycles, finally, 15 s at 95 °C, 1 min at 60 °C and 15 s at 95 °C for 1 cycle. All groups underwent three technical replicates and three biological replicates. The relative expression levels of target genes were obtained by the 2^−ΔΔCt^ method, with glyceraldehyde-3-phosphate dehydrogenase (GAPDH) as an internal reference. The primer sequences are listed in Table 1.

**Table 1:** The sequences of specific primer.

Gene name	Primer sequence (5′ to3′)
GAPDH-F	GGTCGGAGTCAACGGATTTG
GAPDH-R	GGAAGATGGTGATGGGATTTC
THEMIS2-F	CTGTCTTCCTCATCTCTGTAG
THEMIS2-R	AAGGTCCTAAGGTTGTGTTC
TLR1-F	CCTCTAACACTTCACTTGATAC
TLR1-R	TTAACTGACCTTCCTGGATG
RBM47-F	ACACAGTGGGAATGACAG
RBM47-R	ACAGTGGAGCACATGATC
DDX60L -F	AGTGGTAGTGAGCAGAGAT
DDX60L -R	TGGTATTAGGAGATGATAGGTC
PARP12-F	CTTGGAGGAGGTGGTGTA
PARP12-R	GAGCAACGCCTTCTATGA
IL18-F	GCTAGAAAGTATCCTTCGTATG
IL18-R	GAATCCTCCTGATAACATCAAG
SMAD9-F	TACACATCTTGGTCAGTTCA
SMAD9-R	ACTATCAACACGGCTTCC
CIITA -F	CGGAATGAACCACATCTTG
CIITA -R	CGCAATGTCCTTCAGAGA
SAMHD1-F	AGGCTGGTCTCAACTCTT
SAMHD1-R	TTGTATCATTGTGCTGTCTG
ITGB2-F	TAACCTCACCAACCTCAAG
ITGB2-R	AGGATGTCACCAATTAACCA
TLR7-F	GGCTCTGATTCTCCTGTAAT
TLR7-R	AACTCCTGACCTCGTGAT
PRKACB-M-F	GTGGAGAGCGTGAAAGAGTT
PRKACB-AS-F	GACAGATCAATGAAAGAGTT
PRKACB-M/AS-R	TGAGTTGGATTCTCCCATTT
ABI1-M-F	TGAGCCCGGGGCTGGTCCAAT
ABI1-AS-F	GCAATAGAAACTGGTCCAAT
ABI1-M/AS-R	AAGTAGTGGAGGAAGTGGAAG
PPP3CB -M-F	GCAGCTGAACCATCAAACTG
PPP3CB -AS-F	GCTGAACCTACATCAAACTG
PPP3CB -M/AS-R	TCTGAGTATTTGCTCTGATG
CHID1-M/AS-F	CTTCTCCAGCAGCGTCTTTG
CHID1-AS-R	AGCCTGGCCAGCCCTCTGGAC
CHID1-M-R	AACAACCAGGTCCCTCTGGAC
ELF1-M/AS-F	CAGTTTTCCTGGTTCATTGA
ELF1-AS-R	ATACTTGAAGGCCAAGAAGC
ELF1-M-R	TGCTTGTCAGGCCAAGAAGC
FXR1-M/AS-F	CCAGCGAATCTCATCACAGT
FXR1-AS-R	CATCTTTTGCCTAGCCCATT
FXR1-M-R	GCAACTGTGACTAGCCCATT
BCLAF1-M-F	TTTATGTTTACTGTCATCTT
BCLAF1-AS-F	TACATTTTTGCTGTCATCTT
BCLAF1-M/AS-R	AAGTAAAGAACGGGGAGATT
PLEC -M/AS-F	TGTCGCATCCACTGAAGCAG
PLEC -AS-R	TGCAGGATGGGGAGCTGCAGCT
PLEC -M-R	TGAGGGCCAACGAGCTGCAGCT
BNIP2-M-F	CTTGATCAACTTGTTTTATGC
BNIP2-AS-F	CTTCTTCATACTGTTTTATGC
BNIP2-M/AS-R	CGTGTTTAATTTGGCAGAAC
ERG -M/AS-F	CTTTAGTTGCCCTTGGTTCT
ERG -AS-R	TGTGCATGGGGTTATTCCAGG
ERG -M-R	CGGCGCTCAGGTTATTCCAGG

### Other statistical analyses

Principal component analysis (PCA) was performed using the R package factoextra (https://cloud.r-project.org/package=factoextra) to display the clustering pattern of samples based on the top two components. The sequencing data and genomic annotations were visualized with a script we developed in-house (sogen) by normalizing the reads by the tags per million (TPM) of each gene. The pheatmap package in R was used for clustering based on Euclidean distance. Student's t tests were performed to determine the significance of differences between groups.

## Results

### Identification of differentially expressed genes (DEGs) at different stages of atherosclerosis as determined through functional analysis

**Figure 1:**
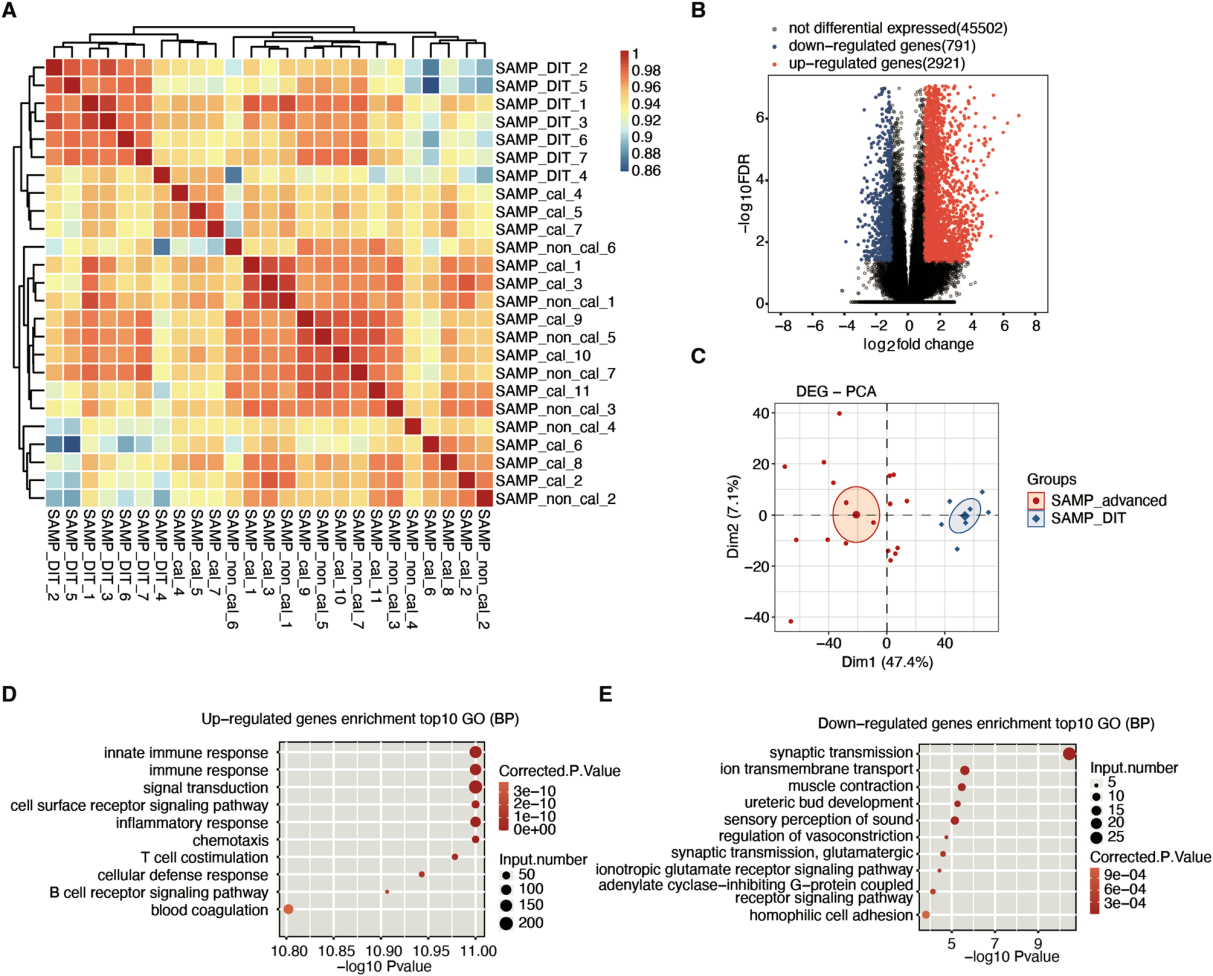
Transcriptome analysis of DEGs in atherosclerotic plaques in the early disease stage (SAMP_DIT) and advanced disease stages (SAMP_advanced). (**A**) Sample correlation between SAMP_DIT and SAMP_advanced (SAMP_cal and SAMP_non_cal) samples.The heatmap scale represent correlation coefficient between different samples. (**B**) Volcano plot displaying DEGs in each group compared to the SAMP_DIT samples. Red indicates genes with upregulated expression (FC ≧ 2 and FDR ≦ 0.05). Blue indicates genes with downregulated expression (FC ≦ 0.5 and FDR ≦ 0.05). (**C**) Principal component analysis (PCA) of 18 SAMP_advanced and 7 SAMP_DIT samples after normalizing all known DEG expression levels. The samples were grouped by left and right atrial appendages and atherosclerotic stage, and the ellipse for each group is a confidence ellipse. (**D**, **E**) GO analysis of DE mRNAs, divided into genes with up- (**D**) and down-regulated (**E**) expression.

IN this study, the RNA-seq data of 32 carotid endarterectomy plaque samples from GSE104140 were downloaded from the GEO database. The data were divided into the very early stage of vascular disease (diffuse intimal thickening) and two advanced stages of atherosclerosis (calcified and noncalcified fibroatheroma formation). We first analyzed samples in each of three different disease stages, and the results showed that the calcified and noncalcified samples contained very few DEGs (Supplementary Fig. 1). Therefore, we combined the calcified and noncalcified samples into one group (SAMP_advanced, advanced stage), and this combined group and the diffuse intimal thickening samples (SAMP_DIT, early stage) were analyzed. The sample correlation analysis also showed that there were significant differences in gene expression between SAMP_DIT and SAMP_advanced. However, the difference between the calcified group and the noncalcified group was smaller (Fig. 1A). A total of 3712 DEGs between the early stage and the advanced stage of atherosclerosis were identified, including 2921 genes with upregulated expression and 791 genes with downregulated expression (Fig. 1B). The PCA with 18 SAMP_advanced and 7 SAMP_DIT samples after normalizing all known DEG expression levels is shown in Fig. 1C. The expression level of all DEGs clearly distinguished the early and advanced atherosclerosis groups, and the samples in the two groups were clustered.

According to GO term enrichment analysis, 2921 upregulated DEGs were significantly enriched in immune-related biological processes, such as the innate immune response, immune response, B-cell receptor signaling pathway and T-cell costimulation pathways. Atherosclerosis is mainly driven by the innate immune response involving myeloid cells, followed by adaptive immunity involving T cells and B cells to modulate the inflammatory state within the plaque, which is the same as the results^15^. Additionally, upregulated DEGs were enriched in “inflammatory response, signal transduction, cell surface receptor signaling pathway, blood agglutination” (Fig. 1D), while “synaptic transmission, ion transmembrane transport, muscle contraction, vasoconstriction regulation” was significantly enriched in 791 downregulated DEGs (Fig. 1E). We also conducted a KEGG pathway analysis, and the five pathways most enriched with DEGs with upregulated expression were cytokine–cytokine receptor interaction, cell adhesion molecules (CAMs) and chemokine signaling pathway, while “cAMP signaling pathway, adrenergic signaling in cardiomyocytes, insulin secretion, long-term potentiation and calcium signaling pathway” were most enriched with DEGs with downregulated expression (Supplementary Fig. 2).

### Regulated alternative splicing events (RASEs) and genes expressed at different stages of atherosclerosis

**Figure 2:**
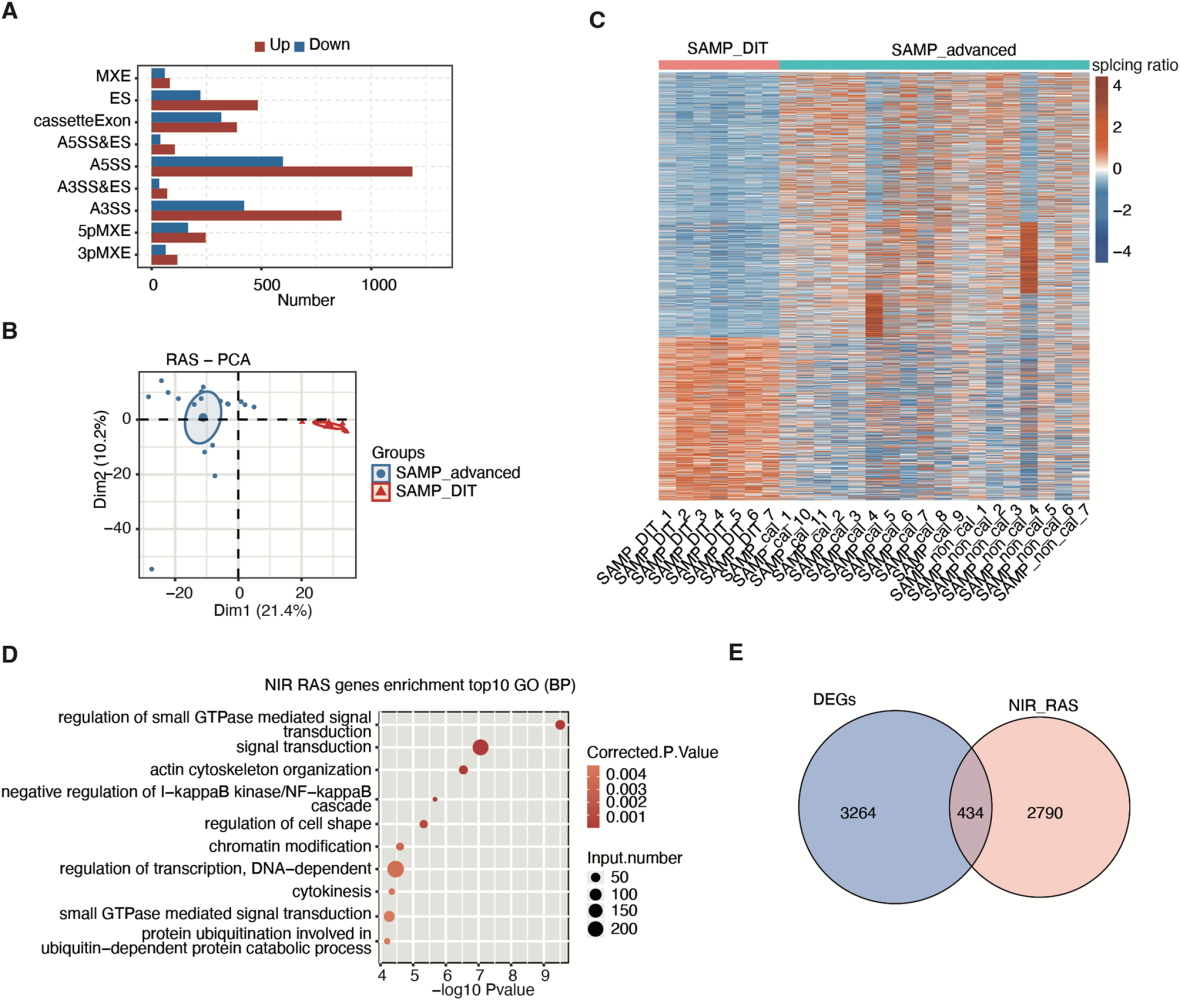
AS deregulation in atherosclerotic plaques in the early disease stage (SAMP_DIT) and advanced disease stages (SAMP_advanced). (**A**) Classification of all RAS events except IR events. X-axis: RAS event number. (**B**) Principal component analysis (PCA) of 18 SAMP_advanced and SAMP_DIT samples based on the percent spliced in (PSI) value of all differential nonintron retention (NIR) events. The samples were grouped by left and right atrial appendages and atherosclerotic stage, and the ellipse for each group is the confidence ellipse. (**C**) PSI heatmap showing all NIR-regulated alternative splicing events (RASEs) among SAMP_advanced and SAMP_DIT samples. NIR RASEs were clustered on the basis of K means. AS filtering was performed to detect splice junctions. AS is indicated if 80% or more of the samples contain 10-splice junction reads. (**D**) A GO analysis of RASGs in the SAMP_ advanced compared with the SAMP_DIT. E. Venn diagram showing the RASGs and differentially expressed genes (DEGs), *p*-value = 1.

We analyzed all RASE events in all samples (Supplementary Fig. 3). Among 5468 RASEs found between the early stage and advanced stage of atherosclerosis, A5SS (alternative 5’ splice site) and A3SS (alternative 3’ splice site) were the most frequently reported (Fig. 2A). A PCA of the 18 advanced samples and 7 DIT samples based on the percent spliced in (PSI) value of all differential nonintron retention (NIR) events shows that the DIT group can be separated from the advanced group (Fig. 2B). A PSI heatmap showing all NIR-RAS events among the SAMP_advanced and SAMP_DIT samples showed obvious differences (Fig. 2C). Compared with the DIT, GO analysis demonstrated that all regulated alternative splicing genes (RASGs) were mainly enriched in atherosclerosis-related pathways like small GTPase-mediated signal transduction, signal transduction, actin cytoskeleton organization, negative regulation of the I-κB kinase/NF-κB cascade, regulation of cell shape, transcription regulation and other functional pathways (Fig. 2D). Small GTPases (ras) activate a variety of downstream signaling pathways, including phosphatidylinositol 3-kinase (PI3K) and Rac and Rho proteins, associated with the regulation of the cytoskeleton. Through RAS, other signals may be activated such as p38 MAPK, stress-activated protein kinase pathway, and c-Jun N-terminal [JNK] pathway^16^. MAPK, JNK pathways and cell migration caused by cytoskeleton changes is closely related to plaque stability^17–19^. In addition, 434 of the 3698 DEGs were involved in nonintron-retained alternative splicing events, suggesting that alternative splicing of genes may play a role in the progression of atherosclerosis. (Fig. 2E).

### Differential expression of RNA-binding proteins (RBPs) and regulated alternative splicing genes (RASGs)

**Figure 3:**
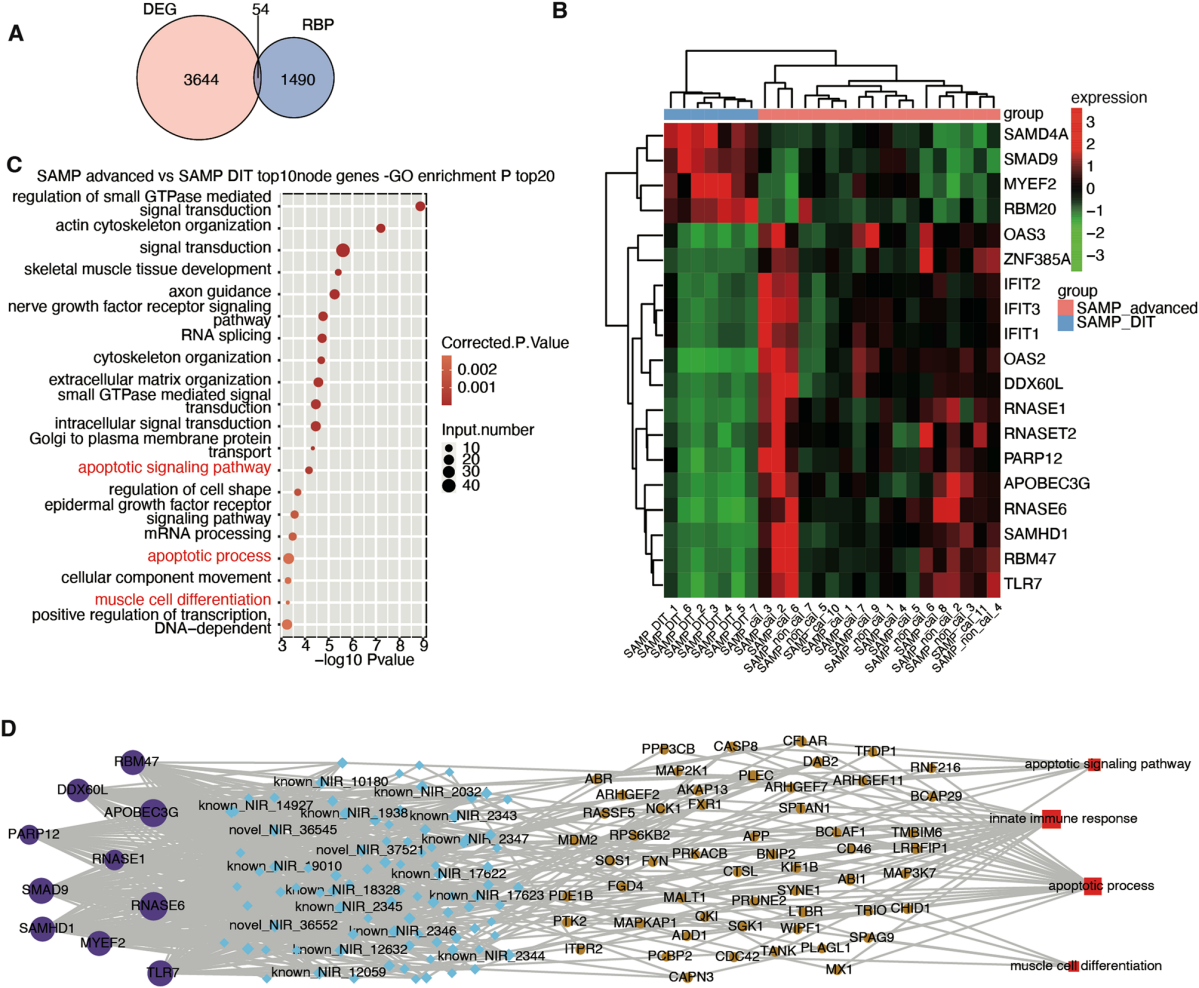
Correlation analysis of RBPs and RASEs in atherosclerotic plaques in the early disease stage (SAMP_DIT) and advanced disease stages (SAMP_advanced). (**A**) Venn diagram depicting regulated differentially expressed genes (DEGs) and RNA binding proteins (RBPs), *p*-value = 1. (**B**) heatmap showing differentially expressed RBPs with a FPKM of greater than 1 in at least 80% of samples. Cluster analysis based on the expression of differential RBP genes between groups. (**C**) The 20 most enriched GO terms in RASGs with expression disrupted by RBPs. (**D**) Deregulation of the AS network by the 10 most enriched RBPs (purple) and RASEs (blue). The 4 enriched GO terms associated with atherosclerotic plaque with RASGs (yellow) with disrupted expression shown in red.

RBPs play important roles in RASEs. Therefore, we explored the relationship between regulated alternative splicing genes (RASGs) and RBPs. we identified 54 differentially expressed RBPs between the two atherosclerotic sample groups(Fig. 3A). Then, a heatmap of these RBPs whose expression levels was greater than 1 in 80% of the samples showed differential expression for the DIT and advanced groups (Fig. 3B). To further demonstrate the regulatory relationship of RBPs on RASGs. We established the regulatory relationship between RBPs and RASGs through correlation analysis and speculated that there may be a potential regulatory relationship. The GO analysis of the highest correlation values ten RASGs that co-regulated with these RBPs was enriched in the regulation of small GTP-mediated signal transduction, RNA splicing, apoptotic signaling pathways, Golgi-to-plasma membrane protein transport, intracellular signal transduction, apoptosis, and muscle cell differentiation categories (Fig. 3C). The KEGG analysis indicated that RBP-regulated ASGs between the two stages of atherosclerosis were enriched in focal adhesion, regulation of actin cytoskeleton, vascular smooth muscle contraction, viral carcinogenesis, mTOR signaling pathway, T-cell receptor signaling pathway, and VEGF signaling pathway (Supplementary Fig. 4). The destruction of focal adhesion signaling leads to the loss of adhesion and apoptosis of endothelial cells^20^. Endothelial cells stimulated by long-term lipids and inflammation secrete cytokines such as VEGF and ICAM^15^, which make smooth muscle migrate actively to the intima of blood vessels, all of which affect the stability of plaque fibrous cap^21^. We also selected high expression and consistency ten RBP genes (TLR7, RBM47, SAMHD1, DDX60L, PARP12, SMAD9, APOBEC3G, RNASE1,RNASE6,MYEF2) for another coexpression analysis to further elucidate the regulatory and interaction relationship between these RBPs and AS. (Fig. 3D). We noted that 55 variable splicing genes including ERG, ELF1, BCLAF1, ABI1, FXR1, CHID1, PLEC, PRKACB, BNIP2, and PPP3CB occurred in co-variation with these ten RBPs, suggesting a potential regulatory relationship between them. In addition, we validated the genes with the highest rate of variable splicing in vitro(Supplementary Fig. 7).The apoptotic signaling pathway, innate immune response, apoptotic process and muscle cell differentiation were revealed by GO analysis of RASGs regulated by RBPs (Fig. 3D). In addition, the coregulation of the AS network demonstrated the regulatory relationship between the 10 most highly expressed RBPs and RASEs (Fig. 3D).

### RNA-binding proteins (RBPs) regulate the alternative splicing (AS) of transcription factor (TF)

**Figure 4:**
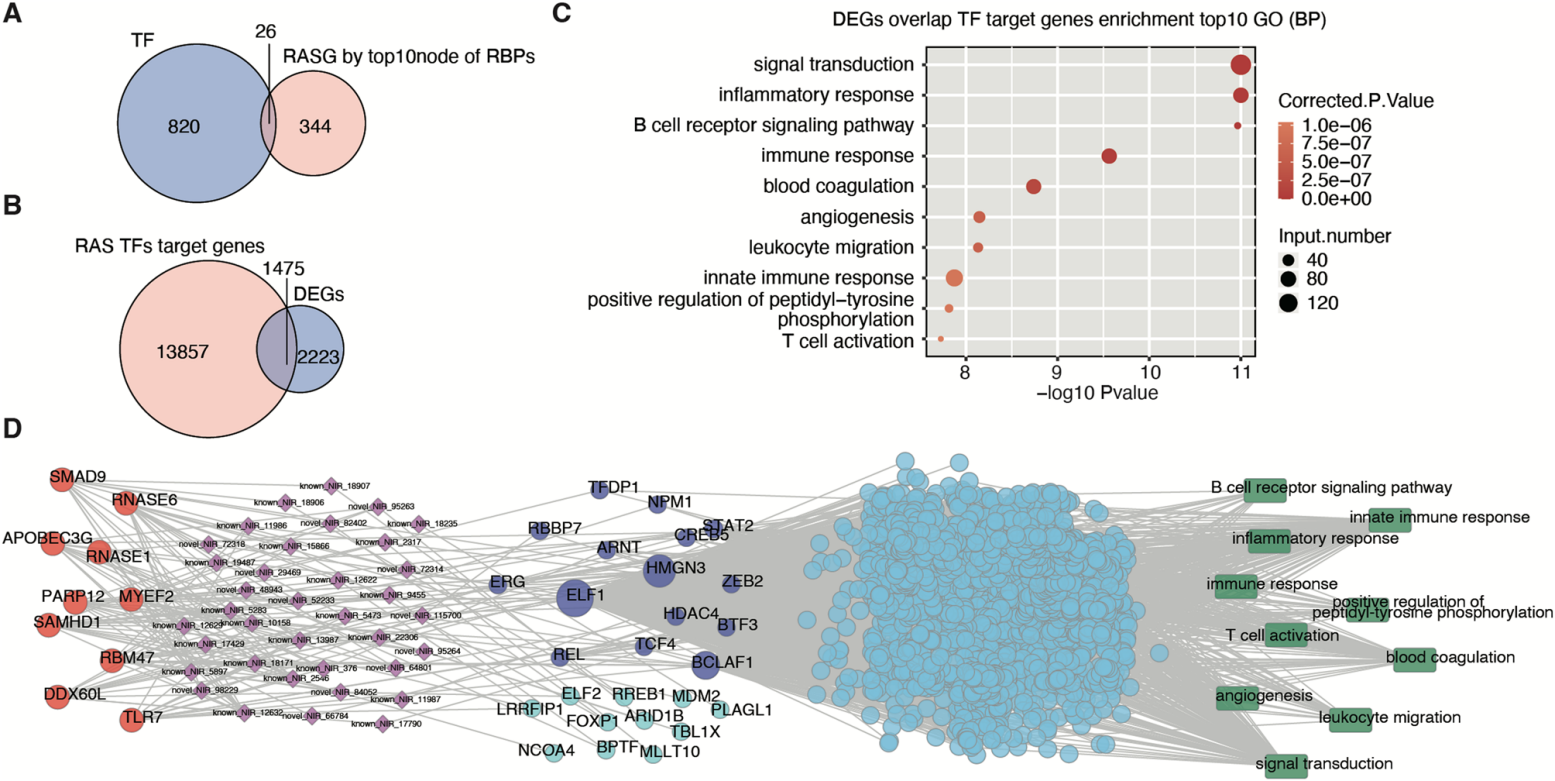
RNA-binding proteins (RBPs) regulate the alternative splicing (AS) of transcription factors (TFs), and differentially expressed target genes of transcription factors (TFs) are enriched in immune and inflammation-related pathways. (**A**) Venn diagram showing TFs and simultaneous deregulation of AS event (ASE) genes, *p*-value = 1 (**B**) Venn diagram showing the overlapping differentially expressed genes (DEGs) and the predicted TF (as shown in Fig. 4A) targets in the Gene Transcription Regulation Database (GTRD; http://gtrd.biouml.org/). The TFs were identified in the GTRD, *p*-value = 1. C The 10 most enriched GO terms as determined by overlapping target mRNAs of the TFs are shown in Fig. 4B. D The simultaneous deregulation of the AS network by the 10 most enriched RBPs (the far left part) and RASEs of TFs (the middle left part). The 10 GO terms most enriched with differentially expressed target genes of TFs (the middle right part) are shown in green.

TFs precisely regulate the transcription process of genes, and many studies have shown that abnormalities in transcription factors are closely related to cardiovascular diseases^22,23^. Therefore, we next explored whether transcription factors were involved in the abovementioned RBP-related alternative splicing events. Among the 344 alternatively spliced genes regulated by the above 10 RBPs, 26 are transcription factors (Fig. 4A). In addition, 1475 target genes of these 26 TFs were differentially expressed (Fig. 4B). We performed a GO analysis with these differentially expressed target genes. The enriched functional pathways were found to be signal transduction, inflammatory response, immune response, leukocyte migration, innate immunity, T-cell activation, coagulation and angiogenesis (Fig. 4C). The KEGG pathway enrichment analysis revealed that crossover genes were enriched in “cytokine‒cytokine receptor interaction, B-cell receptor signaling pathway, cell adhesion molecules, chemokine signaling pathway, leukocyte transendothelial migration, hematopoietic cell lineage” (Supplementary Fig. 5). The AS network deregulated by the 10 most enriched RBPs, RASEs of TFs, differentially expressed target genes of TFs and related pathways is shown in Fig. 4D.

### RBM47 is involved in the regulation of transcription factor (TF) splicing

**Figure 5:**
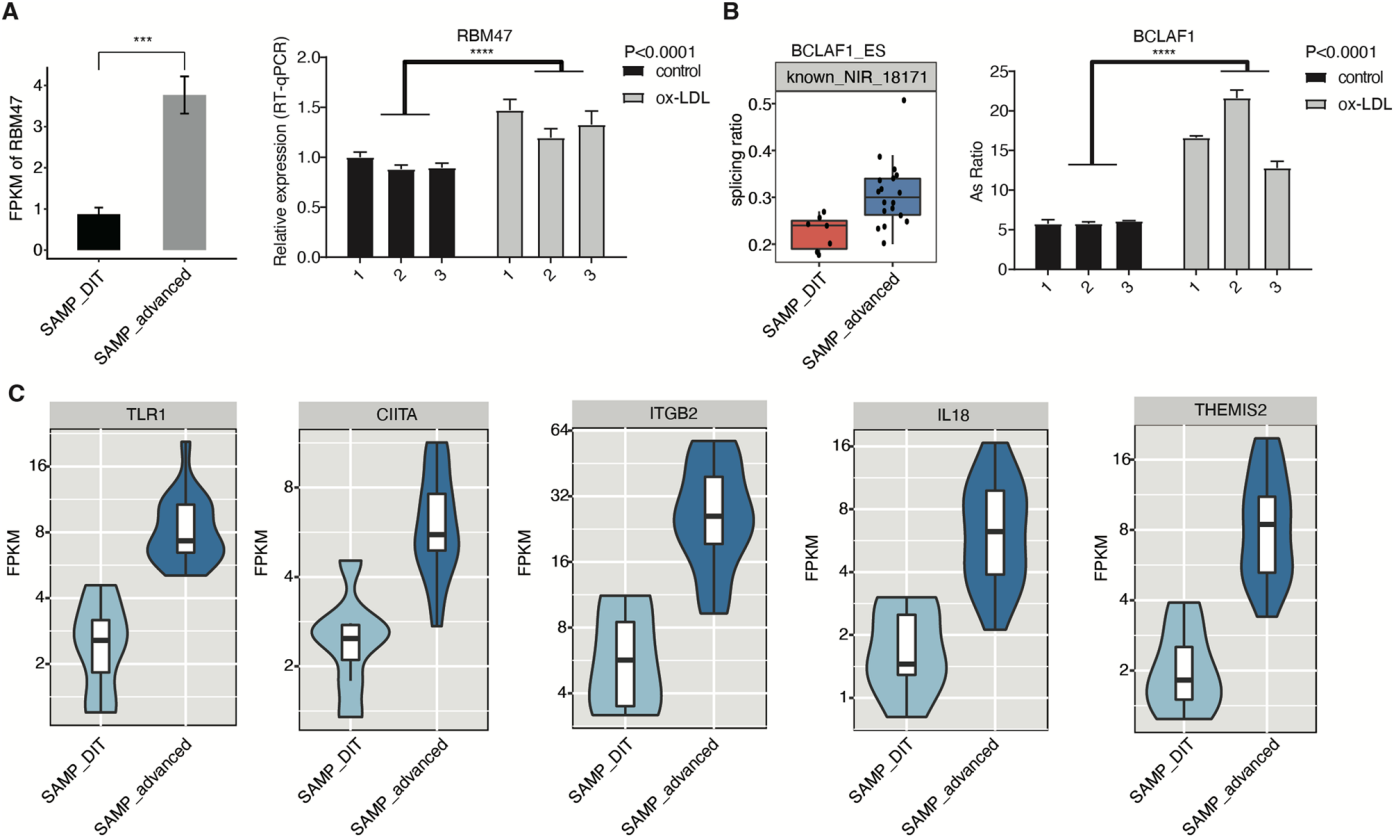
RBM47 is involved in the regulation of transcription factor (TF), which mediates gene expression in inflammatory-related pathways in atherosclerotic plaques. (**A**) The left panel shows RBM47 expression levels in the early disease stage (SAMP_DIT) samples and advanced disease stages (SAMP_advanced) samples. ***: *p* value < 0.001. The right panel demonstrates the expression of RBM47 in three biological replicates of HUVEC stimulated with ox-LDL. ****: *p* value < 0.0001. (**B**) Box plots in the left panel show the splicing ratio of RAS sites located in BCLAF1. The right panel shows the validation of this RASE in three biological replicates of HUVEC stimulated with ox-LDL by RT–qPCR. ****: *p* value < 0.0001. (**C**) Illustration of five differentially expressed target genes that are regulated by ERG, BCLAF1, and ELF1 in the inflammatory pathway.

Among the RBPs noted above, TLR7^24–26^ and RBM47^27^ have been reported to be associated with atherosclerosis, while SAMHD1^28^, DDX60 L^29^, and PARP12^30^ have been reported to be indirectly associated with atherosclerosis. Therefore, we investigated whether these RBPs (TLR7, RBM47, SAMHD1, DDX60 L, PARP12, and SMAD9) were altered in vitro (Supplementary Fig. 6). HUVECs were stimulated with ox-LDL to mimic cholesterol loading of the endothelium in atherosclerosis*.* The results showed that RBM47, TLR7 and SAMHD1 were significantly different between two groups, but only RBM47 was consistent with the RNA-seq data in GSE104140 (Fig. 5A). According to the results shown in Fig. 4D, ERG, ELF1, and BCLAF1 are all TFs that may be regulated by RBM47, so we verified whether alternative splicing occurs in ERG, ELF1, and BCLAF1. The results showed that the percentage of ASEs of BCLAF1 increased, which is consistent with the RNA-seq data in GSE104140 (Fig. 5B). ELF1 has a decreased probability of variable splicing events in the ox-LDL group and ERG was not significantly different in the two groups (Supplementary Figure ). Fig. 5C shows the expression of five differentially expressed target genes in the inflammatory pathway that are regulated by BCLAF1, ERG, and ELF1 which are coregulated with rbm47. These results suggest that RBM47 affects the inflammatory state in atherosclerosis by modulating the AS of BCLAF1.

## Disscussion

Atherosclerosis is a chronic disease of the large and medium arteries that involves multiple factors including lipid metabolism, validation, and oxidative stress., and coronary heart disease, stroke and other diseases caused by atherosclerosis are among the most common causes of death worldwide^3^. Instability and rupture of plaque are the key factors causing cardiovascular and cerebrovascular emergencies. The progression of atherosclerosis and stabilizing plaque remains an unsolved problem.Therefore, we selected gene expression data from very early vascular disease (diffuse intimal thickening) and two advanced atherosclerotic stages. (GSE104140), in order to find possible potential targets for plaque progression. We identified 3712 differential genes, which were mainly enriched in T-cell aggregation-related functional pathway and the regulation of vasoconstriction In the early stage of atherosclerosis, immune cells such as monocytes and leukocytes are recruited to the subendothelium, and oxidatively modified LDL triggers T helper 1 (Th1) cell plaque formation with macrophages^34^. Various TLRs are expressed on the surface of macrophages. Modified LDL and its products may be endogenous ligands of TLR2 and TLR4, and the downstream signaling molecule MY88 is involved in important proatherosclerotic signaling^35^. Activated T cells comprise an important cell population in atherosclerosis, and notably, the incidence of atherosclerosis was lower in T-cell-deficient Apoe − / − mice than in immunocompetent Apoe − / − mice. Transfer of CD4 + T cells into immunodeficient Apoe − / − mice severely exacerbated atherosclerosis^36^.

AS regulation is a major regulatory mechanism leading to proteome diversity, and AS abnormalities have been identified in cardiovascular disease^7^. Although our study showed that A5SS and A3SS were the most prevalent in 5468 differential AS events. However, exon skipping predominated in previous studies of variable splicing in atherosclerosis, like FOXP3 and VEGF165. FOXP3 is a transcriptional regulator that is crucial for the function of regulatory T cells (Tregs)^31^. Treg cells can inhibit plaque progression by regulating the inflammatory response and lipoprotein metabolism^32,33^. Joly et al. found that FOXP3 produces two isoforms through alternative splicing—Full-length FOXP3 (FOXP3fl) and FOXP3 lacking exon 2 (FOXP3Δ2) isoforms^8^. Treg cell activation resulted in increased FOXP3 expression that predominantly was made up of FOXP3Δ2. In a cohort of patients diagnosed with > 70% carotid stenosis, plaques from patients without symptoms were compared with plaques from patients with recent (< 1 month) vascular symptoms(minor stroke, transient ischemic attack, or amaurosis fugax) ,plaque instability was associated with lower FOXP3Δ2 transcripts. However, no differences were found in total FOXP3 mRNA levels^8^.Moreover, Zhao et al. found that VEGF165 has two isoforms. VEGF165 promotes angiogenesis, and VEGF165b inhibits angiogenesis^9^. These studies revealed an association between AS and the development of atherosclerosis.

RBPs has been known to control and regulate the fate of all RNAs within the cell, which includes RNA splicing^34^.Therefore, we analyzed the differential RBPs in the dataset. RBM47,TLR7, RNASE6, SAMHD1, SMAD9, RNASE1, DDX60 L and PARP12 were identified as hub RBPs in plaque progression. RBM47 is a key player in gene posttranscriptional regulation with one or more RNA recognition motif domains^35^ and is involved in the editing of ApoB mRNA, which affects the production of the ApoB functional proteins APOB100 and APOB48^36^. Apolipoprotein B, the cholesterol-carrying component of LDL, is known to have a carcinogenic effect on the retention and accumulation of subendothelial lipids in arteries^37^. Glykeria K et al. found that patients with higher levels of TLR7 transcripts presented with a lower risk of major cardiovascular and cerebrovascular events in the follow-up period after carotid endarterectomy, and carotid plaque immunohistochemistry showed that TLR7 was expressed in all plaques by T cells, macrophages and endothelial cells in capillaries^24^. Similar to TLR7, SAMHD1^38^ initiates innate immunity mainly by binding endogenous ligands, including viral-derived ssRNA and mononucleotides^39^. However, recent studies have pointed to several endogenous ligands, such as RNA from apoptotic cells and extracellular miRNA^40^. In atherosclerosis, RNA from apoptotic and necrotic cells and from tissue damage may be reasonable sources of ligands. It has been shown that hypomethylation of the Rnase6 promoter enhances the proliferation and migration of mouse aortic vascular smooth muscle cells and aggravates atherosclerosis in mice^41^. However, it is not clear whether it affects the disease state in atherosclerosis by exerting RNA enzymatic effects. Smad signaling is important for mediating TGF-β signaling from the cell surface to the nucleus^42^. Upstream BMP4 induces mouse foam cell formation via SMAD9^43^. DDX60 L and PARP12 are related to immune and inflammatory responses that contribute to the development of atherosclerosis ^44^. The endothelial extracellular endonuclease ribonuclease 1 (RNase1) belongs to the ribonuclease A superfamily^45^, which is mainly expressed in various vascular endothelial cells^46^. There are many Weibel-Palade Bodies (WPBs) in the vascular endothelium, which store inflammatory mediators, including RNase1, IL-8, and chemokines^47^. When endothelial cells are activated, eRNA is released. eRNA acts as an immune inducer, enabling WPBs to release inflammatory mediators and trigger inflammation. At this time, the RNase 1 released at the same time can bind to eRNA so that the blood vessel can be protected from a severe inflammatory response^48,49^. The vascular endothelium is the initiating site of atherosclerosis, and good endothelial function and protection from inflammation are important for preventing atherosclerosis. Some RBPs have not been reported to be related to atherosclerosis, such as MYEF2. MYEF2 is implicated in neurodegeneration; MYEF2 is a transcriptional repressor of the myelin basic protein gene and is involved in central nervous system development by controlling oligodendrocyte progenitor cell differentiation through the regulation of myelin protein expression^50^. Atherosclerosis is not only a vascular disease but is also associated with neurological disease^51^. We speculate that atherosclerosis may be related to sympathetic modulation. These differentially expressed RBPs provide a basis for our follow-up study of the RBP-AS-atherogenic regulatory network.

Through joint analysis, several AS genes (ABI1, FXR1, CHID1, PRKACB, and PPP3CB) were found to be simultaneously changed with changes in RBP expression. These AS genes were mainly enriched in RNA splicing, apoptotic processes, and signal transduction, which are related to atherosclerosis. AS abnormalities of these genes may have a direct relationship and play a role in atherosclerosis. AbI1 AS has been reported to contribute to macrophage differentiation from vascular SMCs during atherogenesis through activation of Rac1 expression and the Rac1-NOX1-ROS pathway, leading to an increase in transcription factor Kruppel-like factor 4 (KLF4) cell-like phenotype regulation^52^. Apolipoprotein M is an apolipoprotein that can bind to HDL-related sphingosine 1-phosphate (S1P), which transports S1P to receptors on the endothelium for endothelial protection^53^. FXR1 can upregulate the expression of apolipoprotein M, thereby mediating antiatherosclerosis^54^. CH1D1 belongs to the family of chitinases and chitinase-like proteins. The chitinase-like protein family chitinase and YKL-40 reflect macrophage activation in atherosclerotic plaques^55^, so we speculate that CH1D1 may also play a similar role in atherosclerosis. cAMP-dependent protein kinases or protein kinase A (PKA) are an important class of protein kinases in eukaryotic cells. The catalytic subunit of PKA is encoded by two major genes, PRKACA and PRKACB^56^. The PRKACB gene encodes several splice variants that are expressed in a highly cell- and tissue-specific manner. Among them, Cβ1 is ubiquitously expressed, Cβ2 is enriched in immune cells, while Cβ3, Cβ4 and their abc variants are only expressed in neuronal cells. Loss of Cβ2 can increase the inflammatory susceptibility of macrophages^57^, and Cβ2 transcripts were reduced in the advanced samples of this study, which may cause plaque progression. Angiotensin II-induced vascular smooth muscle exhibits increased PPP3CB-dependent extracellular matrix secretion, suggesting that PPP3CB destabilizes plaques during atherosclerosis^58^.

We analyzed the TFs that underwent alternative splicing to reveal a possible RBP-TF regulatory relationship in atherosclerosis. Notably, Urszula R et al. investigated molecular signatures in human plaques stratified by echogenicity as determined by duplex ultrasound^59^, and BCLAF1, which is similar to ZEB2, was identified as an important gene in atherosclerosis. Moreover, BCLAF1 appears to be functionally required for SMC survival and transdifferentiation to a macrophage-like phenotype^59,60^. The signal transducer and activator of transcription (STAT) family includes STAT1, STAT2, and STAT3, which transmit signals from the proinflammatory cytokines interferon (IFN)α and IFNγ and pattern recognition receptor (PRR) Toll-like receptors, which are found in blood vessels. An inflammatory environment is formed under the vessel membrane to promote atherosclerosis^61,62^. The transcription factor ERG is essential for endothelial homeostasis, driving the expression of lineage genes and suppressing proinflammatory genes, all of which are associated with atherosclerosis^63,64^.

The analysis of differentially expressed RBPs and differential AS events in atherosclerosis in this study will help us to better understand the molecular mechanism of the occurrence and development of atherosclerosis and will provide new directions and ideas for discovering molecular therapeutic targets for atherosclerosis.

## Supplementary Information

Supplementary Figures.

## Data Availability

The datasets analysed during the current study are available in the Gene Expression Omnibus (GEO) repository (https://www.ncbi.nlm.nih.gov/geo/query/acc.cgi?acc=GSE104140).
