# Supplementary material for: Transcriptome-wide analysis reveals the coregulation of RNA-binding proteins and alternative splicing genes in the development of atherosclerosis
Source: Sci Rep. 2023 Jan 31;13:1764. doi: 10.1038/s41598-022-26556-6 (PMC9889815; doi:10.1038/s41598-022-26556-6)
Supplement: Supplementary file 1 — Supplementary Figures. [file 41598_2022_26556_MOESM1_ESM.docx]

Supplementary Material

| DEG group | Total genes | No. of genes with upregulated expression | No. of genes with downregulated expression | Total |
| --- | --- | --- | --- | --- |
| SAMP_non_cal vs. SAMP_DIT | 49214 | 2387 | 686 | 3073 |
| SAMP_cal vs. SAMP_non_cal | 49214 | 2 | 1 | 3 |
| SAMP_cal vs. SAMP_DIT | 49214 | 2740 | 987 | 3727 |

**Supplementary Figure 1. Total number of differentially expressed genes (DEGs) of three different stages of disease.**


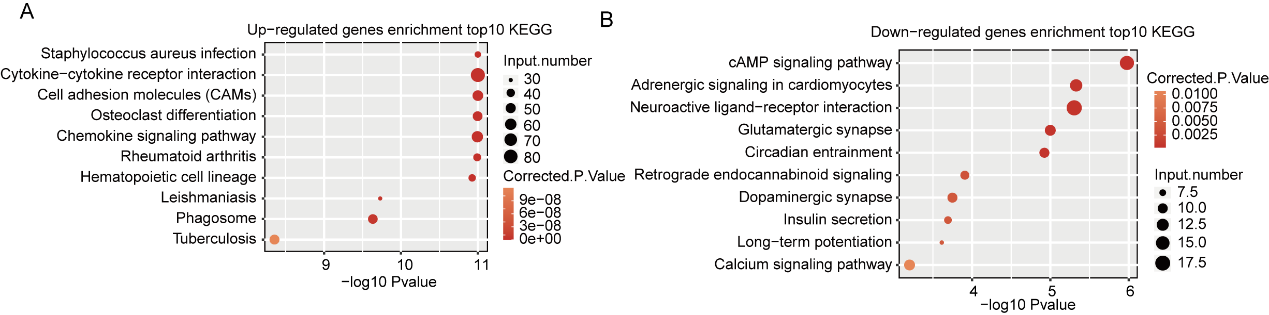


**Supplementary Figure 2. Functional pathways of differentially expressed genes (DEGs) in atherosclerotic plaques in an early disease stage (SAMP_DIT) and advanced disease stages (SAMP_advanced).**

A-B. KEGG analysis showing differentially expressed (DE) mRNAs, categorized into genes with up- (A) and downregulated (B) expression.


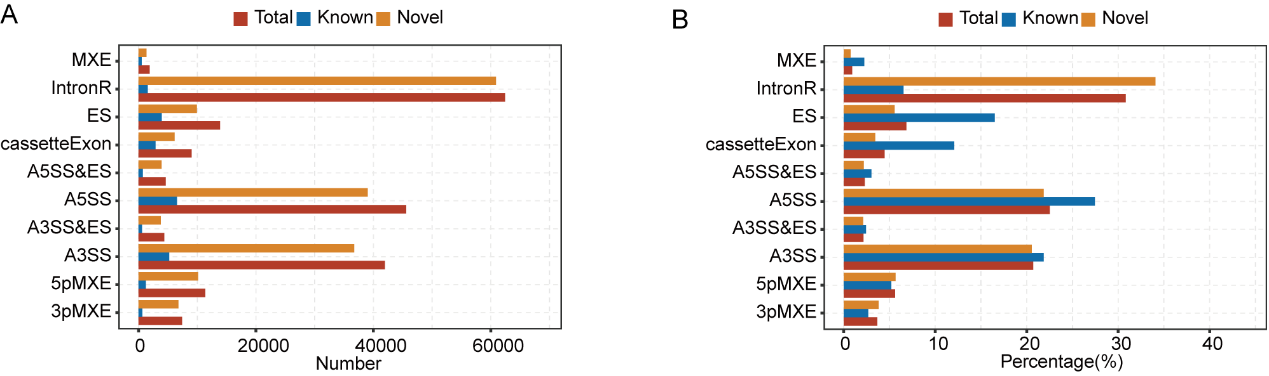


**Supplementary Figure 3. Classification of all the regulated alternative splicing events (RASEs).**

1. Classification of all the RASEs. X-axis: RASE number.
2. Classification of all the RASEs. X-axis: RASE percentage.

**
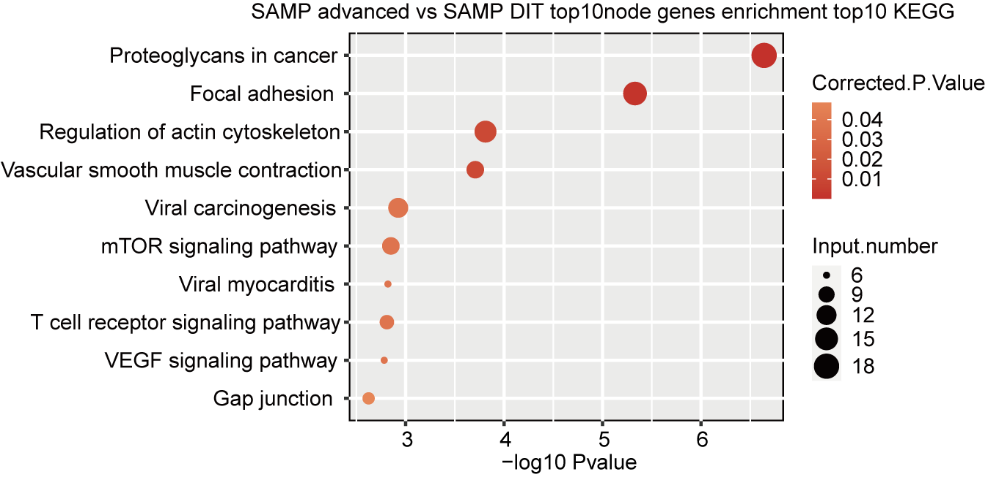
**

**Supplementary Figure 4. KEGG term analysis of regulated alternatively spliced genes (RASGs) in the SAMP_advanced samples compared with the SAMP_DIT samples.**

**
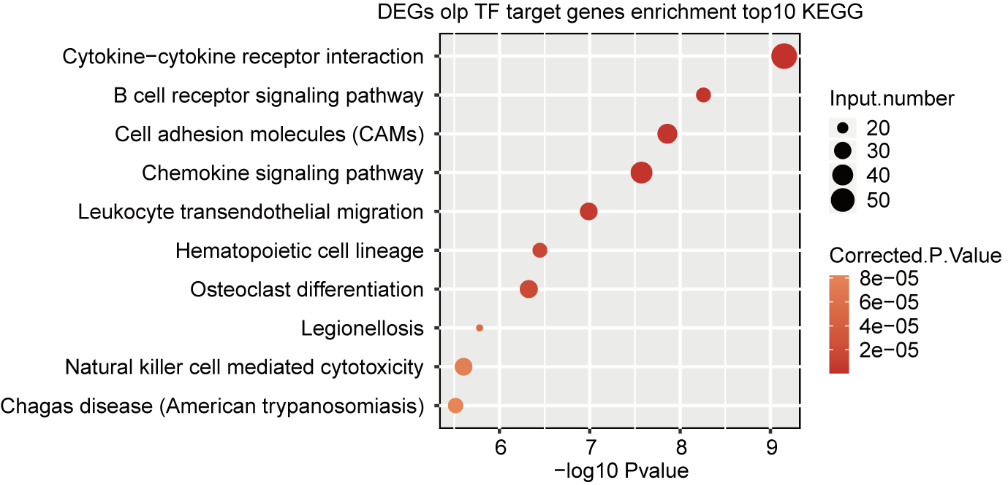
**

**Supplementary Figure 5. The 10 most enriched KEGG terms as determined after overlapping target mRNAs of the transfection factors (TFs) shown in Fig 4B.**


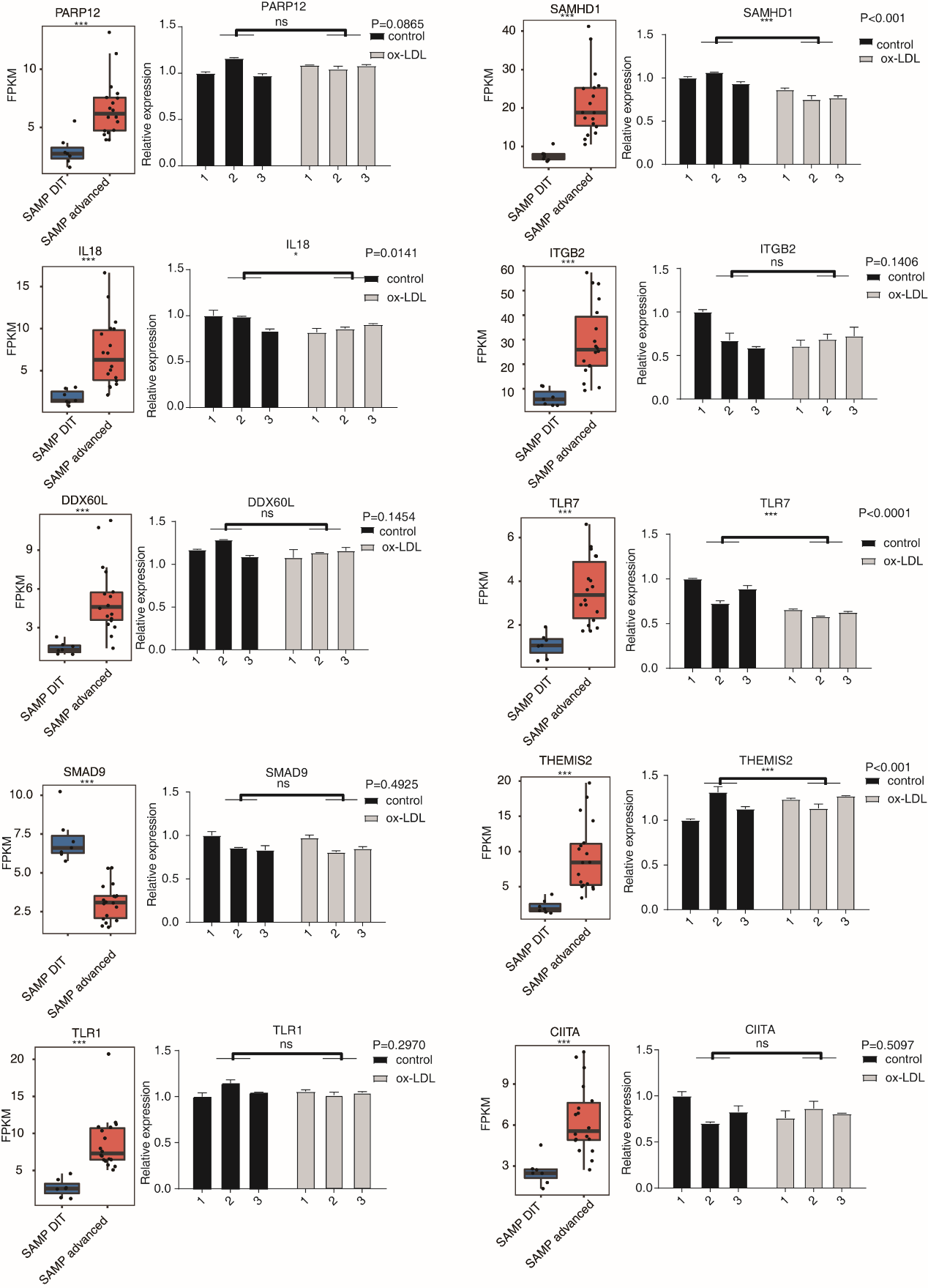


**Supplementary Figure 6：** qRT-PCR was performed to detect differentially expressed 6RBP genes(TLR7、SAMHD1、DDX60L、PARP12、SMAD9) and 5 TFs target genes in HUVECs were exposed to ox-LDL(100 μg/ml) for 24 h. The left panel shows these genes expression levels in the early disease stage (SAMP_DIT) samples and advanced disease stages (SAMP_advanced) samples. The right panel demonstrates the expression of thses genes in HUVEC stimulated with ox-LDL. Each group have three biological replicates. ∗p < 0.05, ∗∗∗p < 0:001, ns: not significant.


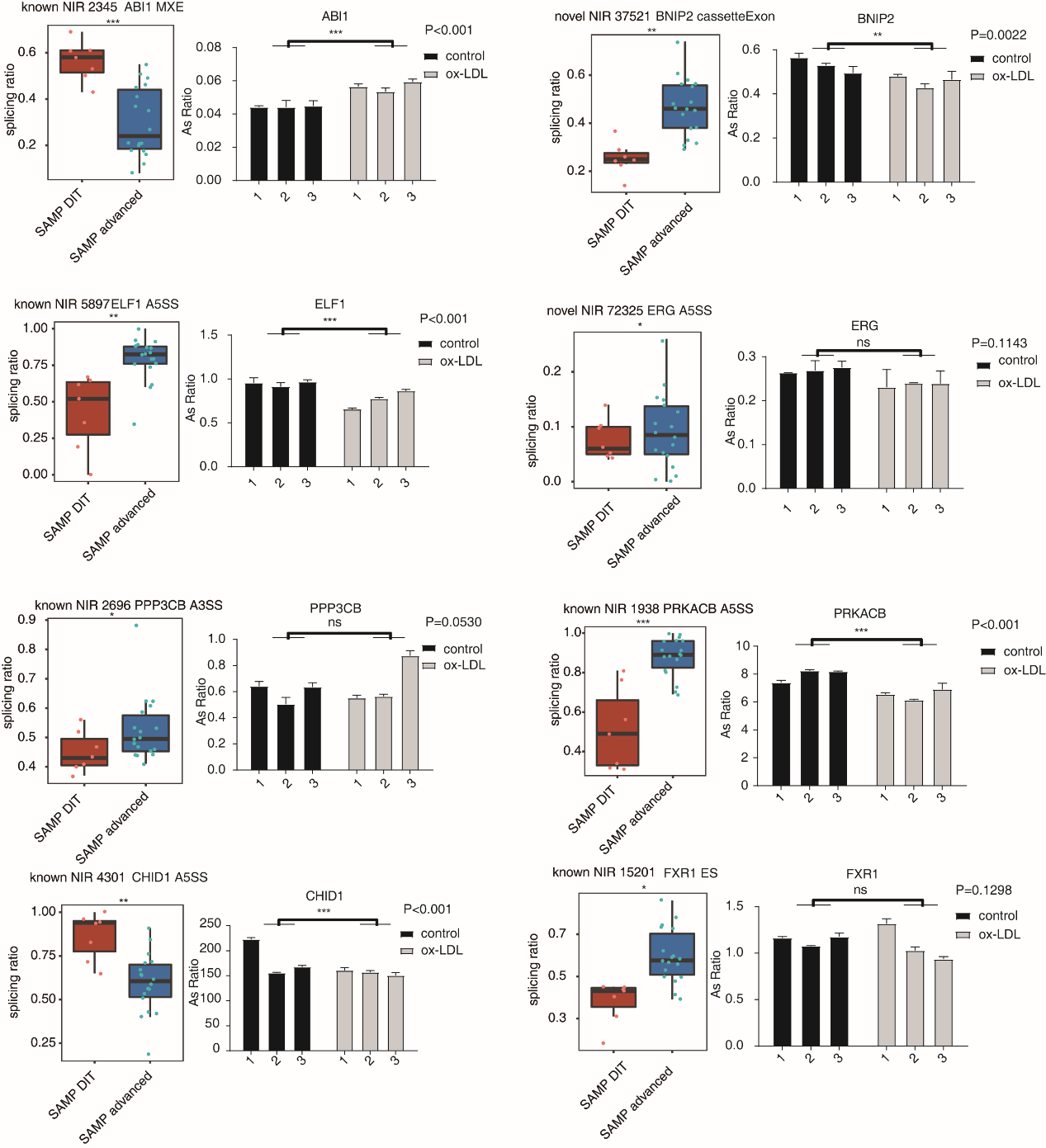


**Supplementary Figure 7：** qRT-PCR was performed to detect splicing ratio of 9 RASG(ERG、ELF1、ABI1、FXR1、CHID1、PLEC、PRKACB、BNIP2、PPP3CB) in HUVECs were exposed to ox-LDL(100 μg/ml) for 24 h. The left panel shows these genes splicing ratio in the early disease stage (SAMP_DIT) samples and advanced disease stages (SAMP_advanced) samples. The right panel demonstrates the splicing ratio of thses genes in HUVEC stimulated with ox-LDL. Each group have three biological replicates. ∗p < 0.05, ∗∗p < 0.01, ∗∗∗p < 0.001, ns: not significant.
